# Irradiation-induced exosomal HMGB1 to confer radioresistance via the PI3K/AKT/FOXO3A signaling pathway in ESCC

**DOI:** 10.1186/s12967-022-03720-0

**Published:** 2022-11-05

**Authors:** Xingyu Du, Xueyuan Zhang, Jing Dong, Naiyi Zou, Dong Guo, Weinan Yao, Xiaobin Wang, Shuguang Li, Chunyang Song, Ke Yan, Wenbin Shen, Shuchai Zhu

**Affiliations:** Department of Radiotherapy, The Fourth Hospital of Hebei Medical University, Shijiazhuang, Hebei People’s Republic of China

**Keywords:** Exosomes, HMGB1, Radioresistance, ESCC

## Abstract

**Background:**

Radioresistance is a major cause of treatment failure in esophageal squamous cell carcinoma (ESCC) radiotherapy, and the underlying mechanisms of radioresistance are still unclear. Irradiation (IR) stimulates changes in tumor-derived exosome contents, which can be taken up by recipient cells, playing an important role in the proliferation, cell cycle and apoptosis of recipient cells. This study investigated the effect of IR-induced exosomal high mobility group box 1 (HMGB1) on radioresistance in ESCC cells.

**Methods:**

Plasma exosomes were isolated from 21 ESCC patients and 24 healthy volunteers, and the expression of HMGB1 was examined. Then, the therapeutic effect of radiotherapy was analyzed according to the different expression levels of plasma exosomal HMGB1 in ESCC patients. The uptake of exosomes by recipient cells was verified by immunofluorescence staining, and the localization of exosomes and HMGB1 in cells before and after IR was evaluated. The effects of IR-induced exosomes on cell proliferation, invasion, apoptosis, cell cycle distribution and radioresistance after HMGB1 knockdown were verified. Moreover, western blotting was used to measure changes in the expression of cyclin B1, CDK1, Bax, Bcl2, phosphorylated histone H2AX and the PI3K/AKT/FOXO3A pathway in the HMGB1-knockdown exosome group and the negative control group.

**Results:**

The expression of HMGB1 in ESCC plasma exosomes was significantly increased compared with that in healthy volunteers, and high expression of HMGB1 in plasma exosomes was associated with radioresistance (P = 0.016). IR-induced the release of exosomal HMGB1 and promoted proliferation and radioresistance in recipient cells, with a sensitization enhancement ratio (SER) of 0.906 and 0.919, respectively. In addition, IR-induced exosomal HMGB1 promotes G2/M phase arrest by regulating the proteins cyclin B1 and CDK1, cooperating with the proteins Bax and Bcl2 to reduce the apoptosis rate through the PI3K/AKT/FOXO3A signaling pathway, and participated in IR-induced DNA damage repair through γH2AX.

**Conclusion:**

These findings indicate that high expression of plasma exosomal HMGB1 is associated with an adverse radiotherapy response. IR-induced exosomal HMGB1 enhances the radioresistance of ESCC cells.

## Background

Esophageal cancer is one of the most common gastrointestinal malignancies in the world, with a high recurrence rate and morbidity, and has become a global public health problem [1]. Esophageal squamous cell carcinoma (ESCC) is the predominant histological classification in China [2]. Although surgery is the most effective treatment for esophageal cancer, most patients are not candidates for direct esophagectomy [3, 4]. For these patients, radiotherapy is an important method to control local recurrence and optimize the surgical strategy [5, 6]. However, local residual lesions often exist, and local control in these patients is poor; the 5-year survival rate of patients that receive radiotherapy alone is only 20.9% [7]. Therefore, identifying new targets to enhance the efficacy of radiotherapy is urgently needed.

Exosomes are vesicular structures produced by multivesicular bodies (MVBs) with diameters ranging from 30 to 150 nm. Exosomes can carry genetic material and biological information between cancer cells and the microenvironment, and play a key role in the establishment of intercellular communication [8, 9]. The released exosomes bind to the receptor on the receptor cell membrane mainly through membrane surface ligands, activating receptor-mediated signal transduction, direct endocytosis of the receptor cell to bring the exosomes into the cell, and direct fusion of the exosome membrane with the cell membrane to bring the contents into the cell, and can change their physiological state [10, 11].

Non-targeting effects can reflect the phenomenon that radiation may indirectly affect non-irradiated cells. In this case, bystander cells receiving stress signals from irradiated cells may exhibit the same properties as irradiated cells. Related studies have shown that radiation can affect the abundance of exosome contents in irradiated tumor cells and share the induction effect of radiation on non-irradiated cells, which may affect the function of recipient cells [12, 13]. A growing number of studies have reported that exosomes contain a variety of biological information, which can be used for biological processes such as cell proliferation [14], angiogenesis [15], invasion or metastasis [16], therapeutic resistance [17], epithelial-mesenchymal transformation, and immune regulation [18], thereby inducing carcinogenesis and even promoting malignant behavior in cancer cells [19, 20].

High mobility group box 1 (HMGB1) is a nonhistone protein that extensively binds to chromosomes in mammalian cells and acts as a molecular DNA chaperone during DNA replication, transcription, recombination and repair [21]. After binding with DNA, HMGB1 regulates gene transcription and maintains the stability of nucleosome structures [22]. When tumor cells are stimulated by physical, chemical, biological and other factors, HMGB1 can be rapidly transferred to the cytoplasm. The C-terminal amino acid residue of HMGB1 is acetylated, and HMGB1 is actively or passively secreted to the extracellular form by triggering lysophosphatidylcholine. These HMGB1-containing exosomes can bind to receptors to stimulate biological effects on target cells [23, 24]. However, the relationship between exosomal HMGB1 and radioresistance in ESCC has not been elucidated.

In this study, we investigated whether plasma exosomal HMGB1 in patients with ESCC was an indicator of the radiotherapy response and the role of irradiation (IR)-induced exosomal HMGB1 in radioresistance.

## Materials and methods

### Clinical serum samples

The study enrolled 21 patients who were admitted to Department of Radiotherapy, the Fourth Hospital of Hebei Medical University (Shijiazhuang, China) from May 2020 to May 2021. Each patient was originally diagnosed with ESCC, and none of them received any prior antitumor treatment, including chemotherapy, radiotherapy, targeted therapy, immunotherapy, or surgical resection. Twenty-four healthy blood donors were recruited from the Physical Examination Center of our hospital. Approximately 1 ml of peripheral blood (EDTA-K2 anticoagulant) was collected from each participant and stored at − 80 °C until use. Treatment response was determined by the RECIST 1.1 (Response Evaluation Criteria in Solid Tumors). This study was approved by the Ethics Committee of the Fourth Hospital of Hebei Medical University.

### ELISA

Plasma concentrations of HMGB1 were measured by using an ELISA kit from CUSABIO (CSB-E08223h, Wuhan, China) according to the manufacturer’s instructions.

### Cell culture and X-ray IR

Human ESCC lines (KYSE150, TE1, KYSE450, KYSE410, KYSE30 and ECA109) were cultured in RPMI-1640 medium supplemented with 10% fetal bovine serum (FBS) and 1% penicillin/streptomycin at 37 °C and 5% CO_2_. All cells were obtained from the Research Center of the Fourth Hospital of Hebei Medical University.

ESCC cells were IR by a 6-MV Siemens linear accelerator (Siemens, Buffalo Grove, IL, USA) at room temperature. The total dose was 6/8/10 Gy, and the dose rate was 300 MU/min. The source-skin distance (SSD) was 100 cm, and cells were then collected at specific times for further study.

### Exosome isolation and identification

When the degree of ESCC cell fusion reached 80%, the medium was replaced with exosome-free fetal bovine serum, and exosomes (Exos) were extracted from the supernatant 48 h later. In addition, the IR group was administered 8 Gy X-ray IR and cultured for 48 h, after which the supernatant was collected to extract exosomes (IR-Exos). Exosomes were carefully extracted from plasma or cell culture supernatant using the exoEasy Maxi Kit (cat. no. 76,064) as described previously [25] and according to the instructions of the manufacturer. All steps were performed at 4 °C. The exosomes were resuspended in sterile PBS and stored at − 80 °C for subsequent experiments. According to the manufacturer’s instructions, a bicinchoninic acid assay (BCA) (Solarbio, Beijing, China) was used to determine the protein concentration of exosomes.

Exosomes were characterized by transmission electron microscopy (TEM), and their morphology was observed. Nanoparticle tracking analysis (NTA) was used to analyze the particle size and collect video images of exosomes. Western blotting was used to measure exosome surface markers (CD9, TSG101, HSP70, and Calnexin).

### Immunofluorescence staining

KYSE150 and ECA109 cells were inoculated on 48-well slides and cultured overnight. The cells were fixed with 4% paraformaldehyde and then permeabilized with Triton X-100 (5%), and nonspecific binding was blocked with a BSA solution. The cells were treated with primary antibodies and incubated overnight at 4 °C. After the unbound primary antibody was removed, fluorescent secondary antibodies (dilution 1:200; cat. nos. SA00013-2 and SA00013-3; Proteintech) were added and incubated at 37 °C for 1 h. DAPI (Solarbio, Beijing, China) was added to the slides for nuclear staining, and glycerol was used to seal the slides. The cells were observed under an ECLIPSE Ti2 confocal laser scanning microscope. The primary antibodies used were anti-HMGB1 (dilution 1:500; cat. no. ab79823; Abcam) and anti-γH2AX (dilution 1:500; cat. no. ab81299; Abcam).

Exos and IR-Exos were fluorescently labeled according to the manufacturer’s instructions. Then, 4 mg/ml DIL solution (Med Chem Express (MCE) Princeton, NJ, USA) was added to PBS containing exosomes and incubates. Exosomes were extracted to remove excess dye. These DIL-labeled exosomes were cocultured with ESCC cells for 24 h, and then the cells were washed with PBS and fixed in 4% paraformaldehyde. Confocal laser microscopy was used to observe the uptake of DIL-labeled Exos and IR-Exos by ESCC cells in both groups. Finally, a laser confocal microscope (Nikon A1, Japan) was used for photography.

### Cell transfection

The HMGB1 knockdown lentivirus was purchased from Gene Chem Co., Ltd. (Shanghai, China). The cells (5 × 10^5^/well) were cultured in 6-well plates until they reached 50% confluence, and then the overexpression lentivirus and the negative control (NC) lentivirus were added and incubated for 24 h according to the instructions. The transfection efficiency was observed by fluorescence microscopy after 72 h. The cells were screened with 1 µg/mL puromycin, subcultured and maintained with 0.25 µg/mL puromycin. After the cells were IR, HMGB1 knockdown exosomes (IR-shHMGB1-Exos) and negative control exosomes (IR-NC-Exos) were extracted from the supernatant after 48 h of culture in exosome-free fetal bovine serum.

### Real-time quantitative polymerase chain reaction (RT‒qPCR)

RNA extraction and RT‒qPCR was performed as described in a previous study [26]. The primers for RT‒qPCR was as follows:

HMGB1:

5′-AATACGAAAAGGATATT GCT-3′ (forward),

5′-GCGCTAGAACCAACTTAT-3′ (reverse);

and GAPDH: 5′-CGCTGAGTACGTCGTGGAGTC-3′ (forward),

5′-GCTGATGATCTTGAGGCTGTTGTC-3′ (reverse).

### Cell counting Kit-8 (CCK-8) assay

KYSE150 and ECA109 cells were treated with exosomes (20 µg/ml) for 24 h, counted and diluted to 2 × 10^4^ cells/ml. A 100 µl cell suspension was inoculated in each well of 96-well plates with 5 replicates per sample. When the cells had adhered, they were IR. Then, 10 µl/well cell counting kit-8 reagent (Med Chem Express (MCE) Princeton, NJ, USA) was added at 24 h, 48 h, 72 h, and 96 h. The absorbance was measured at 450 nm by Multiskan 2 h later. The experiment was repeated three times before analysis.

### Colony formation assay

KYSE150 and ECA109 cells were IR with 0, 2, 4, 6 and 8 Gy, and the appropriate cells were treated with exosomes (20 µg/ml) for 24 h and plated on 6-well plates with 3 replicates in each group. After 14 days, the cells were fixed with paraformaldehyde (4%) and stained with crystal violet (0.1%). A colony count (> 50 cells) was then performed. The results were analyzed by Graph Pad Prism 8.0 (Graph Pad Software, Inc., La Jolla, CA, USA) to calculate survival curves (SF), and the multitarget single-hit model was fitted to the data using the formula [SF = 1 − (1 − e−D/D0) N].

### Flow cytometry

Flow cytometry (FCM) was used to observe the effect of exosomal HMGB1 after IR on the cell cycle and apoptosis. The cells were collected according to the instructions after 48 h. The cells were stained with a propidium iodide solution (Multi Sciences Biotech Co., Ltd.) to detect cell cycle distribution. Annexin V and propidium iodide (BD Biosciences, San Jose, CA, USA) were used to measure the apoptosis rate.

### Western blotting

The procedures were conducted as previously described [27]. The membranes were scanned, and the relative grayscale value of each protein was examined by an Odyssey Infrared Imaging System (LI-COR Biosciences, Lincoln, NE, USA). The density of the protein bands was semiquantified using ImageJ (National Institutes of Health, Bethesda, MD, USA). To observe the changes in the protein expression levels, we calculated the ratio of each protein to the corresponding GAPDH / β-actin level. Three separate experiments were conducted for each western blotting analysis. The antibodies used were the exosome panel (Calnexin, CD9, HSP70, TSG101; dilution 1:1000; cat. no. ab275018; Abcam), anti-HMGB1 (dilution 1:10,000; cat. no. ab79823; Abcam), anti-γH2AX (dilution 1:1,000; cat. no. ab81299; Abcam), anti-Cyclin B1 (dilution 1:50,000; cat. no. ab32053; Abcam), anti-CDK1 (dilution 1:10,000; cat. no. ab133327; Abcam), anti-Bax (dilution 1:5000; cat. no. 60267-1-Ig; Proteintech), anti-Bcl2 (dilution 1:2000; cat. no. ab182858; Abcam), anti-GAPDH (dilution 1:5000; cat. no. 60004-1-Ig; Proteintech), anti-β-Actin (dilution 1:5000; cat. no. 60009-1-Ig; Proteintech), anti-p-AKT (dilution 1:2000; cat. no. 66444-1-Ig; Proteintech), anti-AKT (dilution 1:1000; cat. no. 4685; Cell Signaling Technology, Inc.), anti-PI3K (dilution 1:500; cat. no. 20584-1-AP; Proteintech), anti-FOXO3A (dilution 1:1000; cat. no. 66428-1-Ig; Proteintech), and anti-p-FOXO3A (dilution 1:1000; cat. no. ab154786; Abcam).

### Statistical analysis

The data are presented as the means ± standard error of measurement (SEM) of at least three independent experiments. Two groups were compared using Student’s t-test (normally distributed data) or the Mann-Whitney U test (nonnormally distributed data). Analysis of variance was used for comparisons between groups of continuous variables. ROC curve analysis was used to compare the predictive ability of plasma exosome HMGB1 levels in ESCC patients for immediate efficacy. A value of *P* < 0.05 was considered statistically significant. Statistical analyses were performed using GraphPad 8.0 Prism software (GraphPad Software, Inc., La Jolla, CA, USA).

## Results

### Elevated plasma levels of exosomal HMGB1 correlate with radioresistance in ESCC patients

**Fig. 1:**
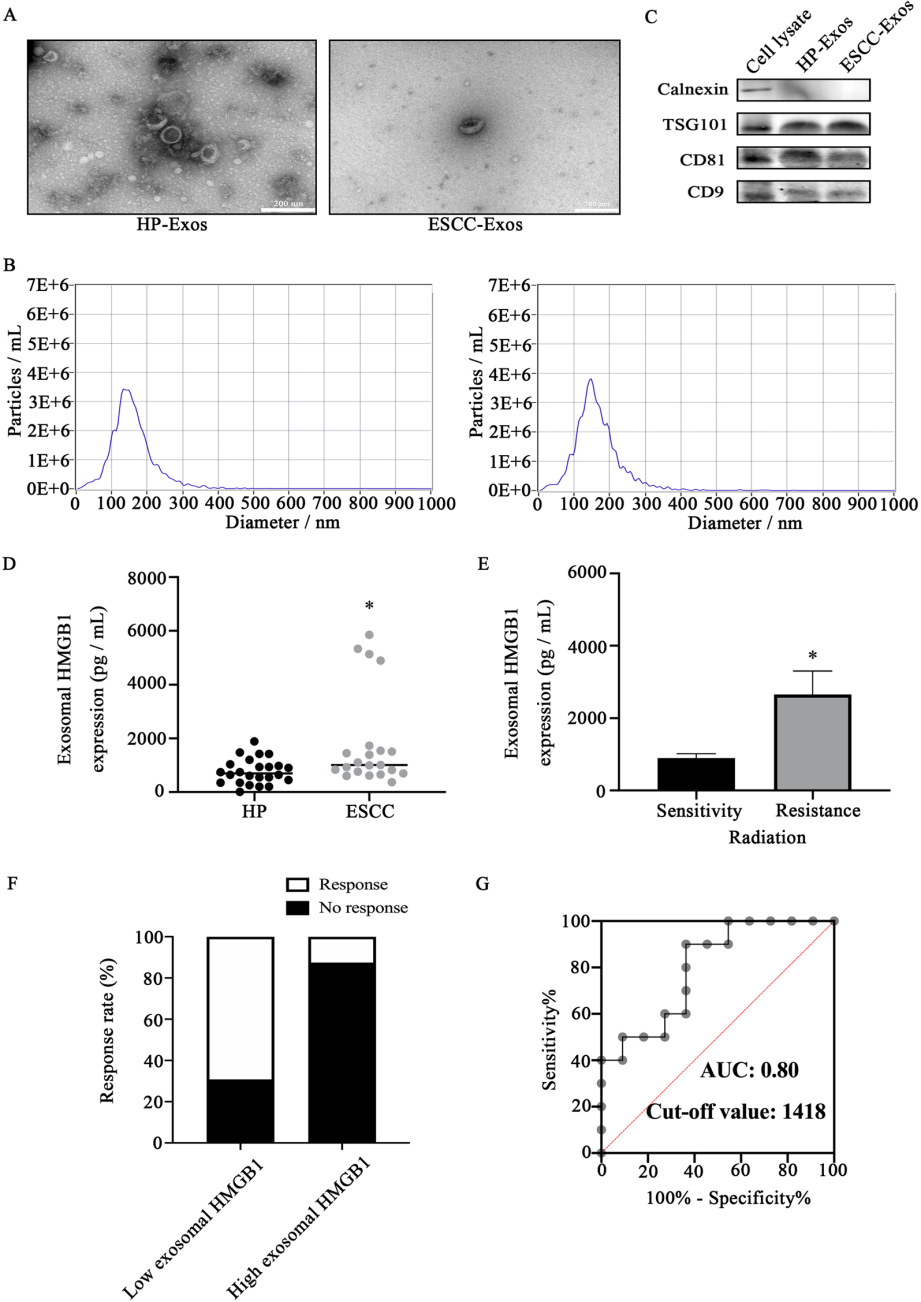
Plasma exosomes characterization and relationship between exosomal HMGB1 and clinical response to radiation. **A** Transmission electron micrograph of HP-Exos and ESCC-Exos. **B** The size distribution and the concentration of the exosomes were measured with Nanoparticle Tracking Analysis. **C** Western blotting of exosomes and cell lysate proteins was performed to confirm exosome marker proteins (CD9, CD81, TSG101) and calnexin. **D** The expression level of HMGB1 in plasma exosomes of ESCC was higher than that of HP. **E** The expression level of exosomal HMGB1 in plasma correlates with ESCC radiotherapy’s efficacy. **F** Response to radiotherapy with different expression levels of exosomal HMGB1. Patients with high exosomal HMGB1 expression showed lower response rate to radiation (P = 0.005). **G** The diagnostic power of plasma exosomal HMGB1 levels for the clinical response to radiation in patients with ESCC. The AUROC of plasma exosomal HMGB1 for clinical response to radiation was 0.80 (95% CI 0.6105–0.9895, P = 0.0201). *P < 0.05; HMGB1: high mobility group box 1; ESCC: esophageal squamous cell carcinoma; HP: healthy people; Exos: exosomes

To investigate the role of exosomal HMGB1 in the response to radiotherapy, plasma samples were obtained from 24 HP (healthy people) and 21 ESCC patients. Transmission electron microscopy (TEM) images showed that plasma exosomes from both groups were spherical and membrane-bound vesicles (Fig. 1A). Nanoparticle tracking analysis (NTA) confirmed that the diameter of exosomes isolated from HP and ESCC plasma was 141.9 and 146.1 nm, respectively (Fig. 1B). Western blotting showed abundant plasma exosome-specific markers (CD9, CD81, TSG101) and the absence of Calnexin (Fig. 1C).

The profile of exosomal HMGB1 was examined by ELISA, and the results revealed that the plasma exosomal HMGB1 levels of ESCC patients (1821 ± 386.6 pg/ml) were considerably higher than those of healthy controls (868.7 ± 119.3 pg/ml) (P = 0.0169) (Fig. 1D). All ESCC patients in this study received intensity-modulated radiotherapy (IMRT), and the response to radiotherapy was evaluated. The expression level of plasma exosomal HMGB1 is closely related to the response to radiotherapy, and the level of exosomal HMGB1 in patients with radioresistance (SD, PD) is higher than that in patients with radiosensitivity (PR, CR) (P = 0.0197, Fig. 1E). Radiotherapy sensitivity included partial response (PR) or complete response (CR) in 12 patients (57.1%), and radiotherapy resistance included stable disease (SD) or progressive disease (PD) in 9 patients (42.9%) (Fig. 1F). The optimal cutoff value of exosomal HMGB1 for predicting the response to radiotherapy was determined by ROC analysis. The results showed that the optimal cutoff value of exosomal HMGB1 was 1418, with an AUC of 0.80 (95% CI 0.6105–0.9895, P = 0.0201, Fig. 1G).

### Characterization of exosomes in the cell supernatant

**Fig. 2:**
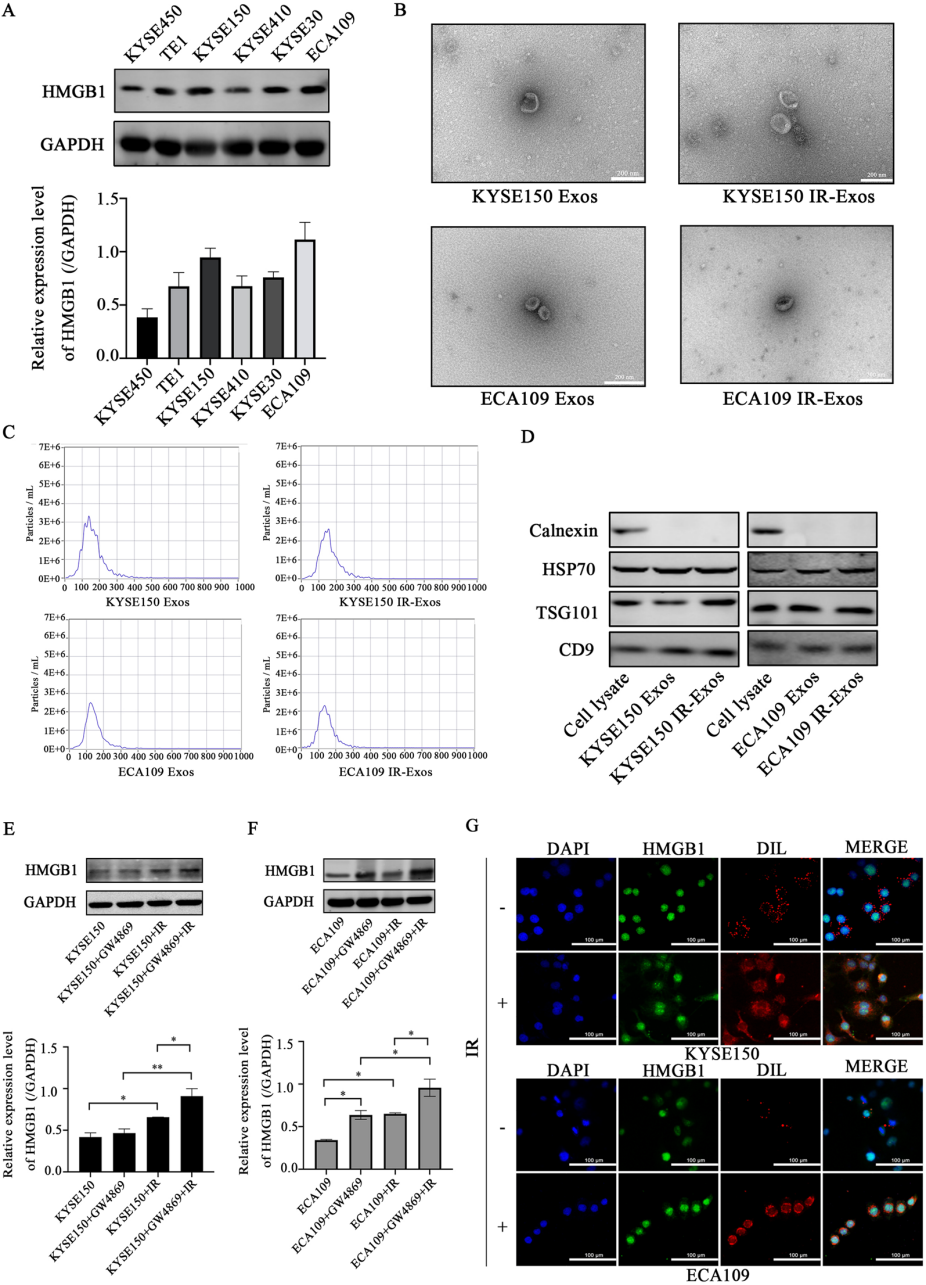
Characterization of exosomes and the effects of radiation on exosomes phagocytosis by KYSE150 and ECA109. **A** The expression of HMGB1 in ESCC cell lines. **B** Transmission electron micrograph of Exos and IR-Exos. **C** The size distribution and the concentration of the exosomes were measured with Nanoparticle Tracking Analysis. **D** Western blotting of exosomes and cell lysate proteins was performed to confirm exosome marker proteins (CD9, TSG101, HSP70) and calnexin. **E**, **F** Changes of HMGB1 protein in KYSE150 and ECA09 after GW4869. **G** DIL (red)-labeled exosomes were co-cultured with KYSE150 and ECA109 for 24 h. Then the phagocytosis of exosomes and the HMGB1(green) position changes before and after irradiation were observed (DAPI-labeled nuclei were blue, magnification, ×600). *P < 0.05; **P < 0.01; IR: irradiation; HMGB1: high mobility group box 1; Exos: exosomes

The expression of HMGB1 in the KYSE450, TE1, KYSE150, KYSE410, KYSE30, and ECA109 ESCC cell lines was determined by western blotting analysis. The expression levels of HMGB1 in KYSE150 and ECA109 cells were significantly higher than those in other cell lines (Fig. 2A). Therefore, KYSE150 and ECA109 cells were selected for further study. The morphological characteristics of exosomes, size distribution and the presence of exosome markers were evaluated by TEM, NTA and western blotting. Exosomes from both groups were observed as round-shaped structures by TEM (Fig. 2B). The NTA results showed that the mode diameters of KYSE150 and ECA109 exosomes were 137.5 nm and 125.0 nm, respectively, and the concentrations were 3.3 × 10^6^ particles/ml and 2.5 × 10^6^ particles/ml, respectively. In contrast, the IR-induced mode diameters were 145.1 nm and 132.4 nm, respectively, with concentrations of 2.5 × 10^6^ particles/ml and 2.3 × 10^6^ particles/ml (Fig. 2C). Both exosome types were characterized by the expression of known biomarkers (CD9, TSG101, HSP70) and the absence of Calnexin (Fig. 2D). These results indicate that there were no significant differences in exosome mass, particle number, or structure before and after IR.

### IR promotes the release of HMGB1 from exosomes

**Fig. 3:**
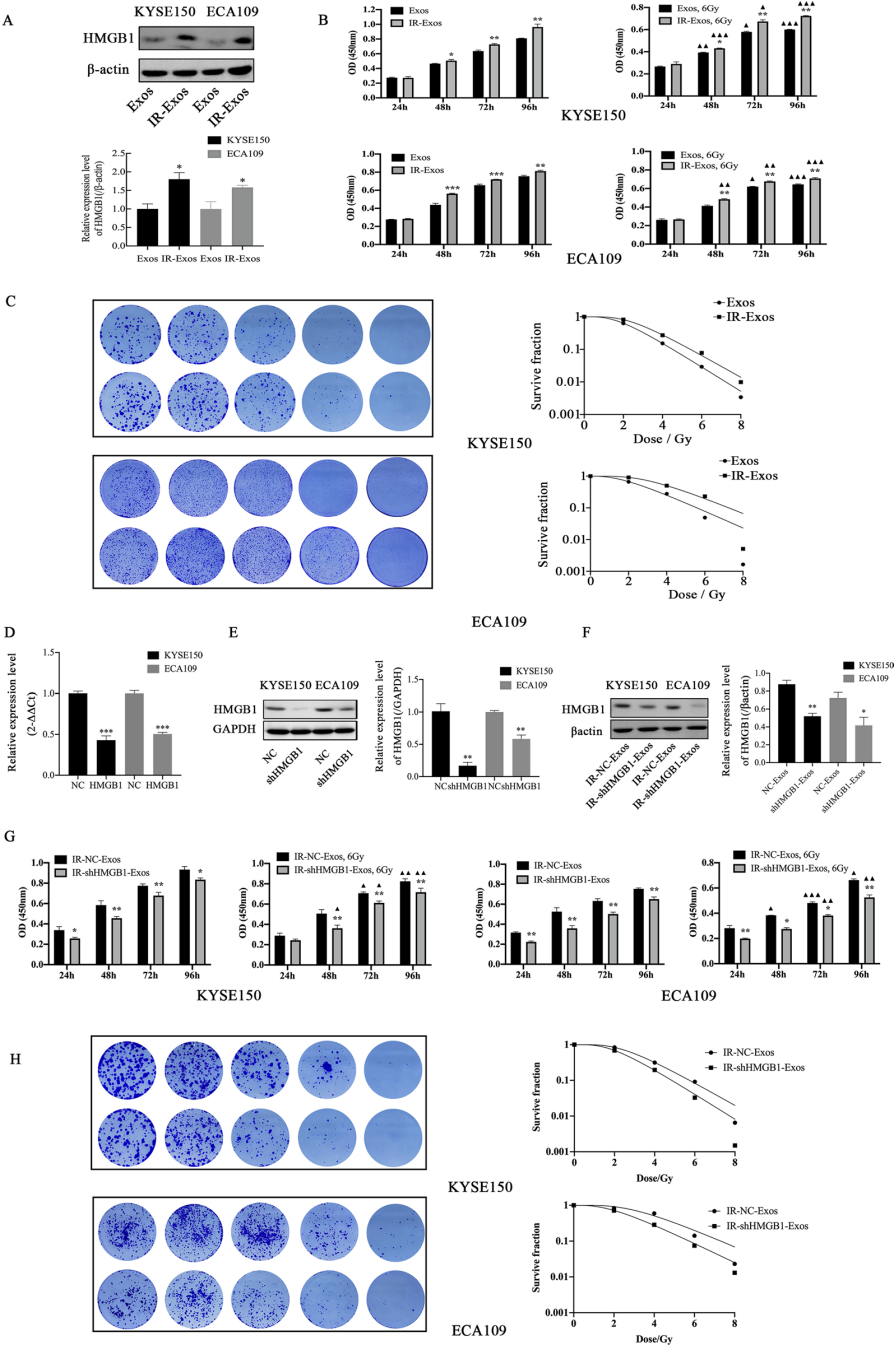
The effect of exosomes on ESCC cells. **A** Radiation-induced changes of HMGB1 in exosomes. **B** The CCK8 assay detected cell proliferation. **C** The radiosensitivity of ESCC cells was measured by clone formation assay. **D** At the mRNA level, HMGB1 mRNA expression in the HMGB1 group was obviously lower than that in the NC group. **E** HMGB1 knockdown cell lines KYSE150 and ECA109 were successfully constructed at the protein level. **F** The expression of HMGB1 in exosomes of knockdown cell lines was significantly lower than that of the NC group. **G** Cell proliferation was detected by CCK8 assay. Cell viability in the IR-shHMGB1-Exos group was lower than that in the IR-NC-Exos group. **H** The radiosensitivity of ESCC cells was measured by a clone formation assay. IR-shHMGB1-Exos group alleviated radioresistance. *P < 0.05, **P < 0.01 and ***P < 0.001; a comparison with the corresponding non-irradiated group is indicated by triangles ^▲^P < 0.05, ^▲▲^P < 0.01 and ^▲▲▲^P < 0.001. CCK-8: Cell Counting Kit-8; HMGB1: high mobility group box 1; IR: irradiation; ESCC: esophageal squamous cell carcinoma; Exos: exosomes

IR-induced the expression of HMGB1 in KYSE150 (P = 0.0387) and ECA109 (P = 0.025) cells, and the intracellular expression of HMGB1 was significantly increased after treatment with GW4869, which weakened the effect of IR-induced HMGB1 release to the extracellular in KYSE150 (P = 0.0379) and ECA109 (P = 0.0186) cells (Fig. 2E, F). The uptake of exosomes into cells was confirmed by staining exosomes with the fluorescent dye DIL. Immunofluorescence analysis was used to observe the expression and localization of HMGB1 and exosomes in KYSE150 and ECA109 cells (Fig. 2G). The results showed that HMGB1 was mainly expressed in the nucleus and exhibited green fluorescence. The green fluorescence moved to the cytoplasm at 2 h after IR, which indicated that IR can induce the release of HMGB1 from the nucleus to the extracellular space. Exosomes showed red fluorescence and colocalized HMGB1. In addition, the IR-induced increase of HMGB1 expression in KYSE150 and ECA109 exosomes was verified by Western blotting (Fig. 3A). Therefore, exosomes may be an important pathway for IR-induced migration of HMGB1.

### IR-induced exosomes promote proliferation and radioresistance

To investigate the effect of IR-induced exosomes on the viability of ESCC cells, cells exposed to different conditions were collected at 24, 48, 72 and 96 h after transfection and IR. The CCK-8 assay results indicated that the proliferation rates in the IR-Exos group were significantly higher at 48 h, 72 and 96 h than those in the Exos group with or without IR (P < 0.05) (Fig. 3B). At 48, 72 and 96 h after IR treatment, cell proliferation in each group decreased to different degrees. In addition, a colony formation assay was used to examine the effect of IR-induced exosomes on the radiosensitivity of KYSE150 and ECA109 cells. The cell survival curve was drawn according to the number of clones formed, and the radiobiological parameters of each group were used for statistical analysis. The results revealed that the radiosensitivity of the IR-induced exosome group was higher than that of the exosome group (Fig. 3C).

### HMGB1 is downregulated in HMGB1shRNA cell lines

Two ESCC cell lines, KYSE150 and ECA09, were transfected with HMGB1-shRNA lentivirus to construct stable HMGB1 knockdown cell lines. RT‒qPCR (Fig. 3D) and western blotting (Fig. 3E) showed that HMGB1‑shRNA significantly downregulated HMGB1 expression in KYSE150 and ECA109 cells. HMGB1 knockdown group (IR-shHMGB1 - Exos) showed significantly lower HMGB1 expression in IR-induced stable HMGB1 knockdown cell line exosomes than NC group (IR-NC-Exos) (Fig. 3F).

### IR-shHMGB1-Exos inhibit proliferation and increase radiosensitivity

The CCK-8 assay results indicated that the proliferation rates in the IR-shHMGB1-Exos group were significantly lower at 48 h, 72 and 96 h than those in the IR-NC-Exos group with or without IR (P < 0.05) (Fig. 3G). Similarly, at 48, 72 and 96 h after IR treatment, cell proliferation in each group decreased to varying degrees. In addition, a colony formation assay was performed to examine the effect of altering HMGB1 expression in exosomes on the radiosensitivity of KYSE150 and ECA109 cells. According to the survival curve, the radiosensitivity of the IR-shHMGB1-Exos group was higher than that of the IR-NC-Exos group (Fig. 3H).

### IR-shHMGB1Exos increase the apoptosis rates of ESCC cells after IR

**Fig. 4:**
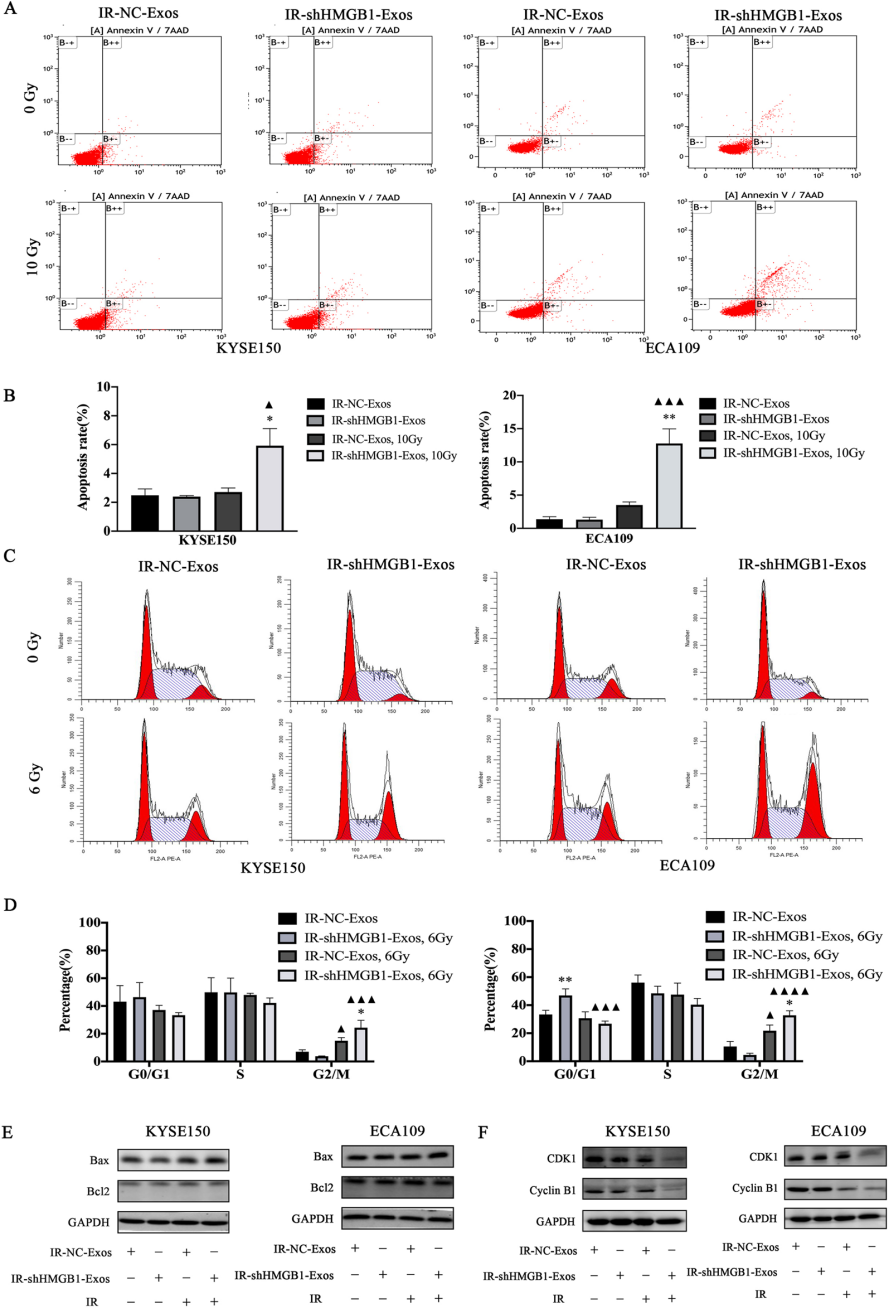
Exosomes regulated the apoptosis rate and cell cycle distribution of KYSE50 and ECA109 ESCC cells. **A**, **B** The apoptosis rate in each group was detected by flow cytometry. **C** IR-shHMGB1-Exos induces G2/M phase arrest in KYSE150 and ECA109 ESCC cells after IR. **D** The histogram of cell cycle distribution was expressed as mean ± standard error for each group. **E** After IR, the proteins Bax/Bcl2 were regulated by both exosomes in KYSE150 and ECA109 cells. **F** IR-shHMGB1-Exos regulated CDK1/Cyclin B1 to induce G2/M phase arrest after IR. A comparison with the IR-NC-Exos group is indicated by asterisks *P < 0.05 and **P < 0.01; A comparison with the corresponding non-irradiated group is indicated by triangles ^▲^P < 0.05, ^▲▲▲^P < 0.001 and ^▲▲▲▲^P < 0.0001. HMGB1: high mobility group box 1; IR: irradiation; NC: negative control; Exos: exosomes

IR-induced apoptosis is one of the mechanisms by which IR kills tumor cells. The changes in cell apoptosis induced by IR were observed by flow cytometry. The apoptosis rate in the IR-shHMGB1-Exos group of KYSE150 (P = 0.033) and ECA109 (P = 0.0021) was higher than that in the IR-NC-Exos group after 10 Gy IR, as evidenced by Annexin V and 7-AAD staining (Fig. 4A, B). Moreover, western blotting was performed to examine the changes in apoptosis-related protein levels after IR. The results showed that compared with those in the IR-NC-Exos group, the expression of Bcl-2 was decreased and the levels of Bax were increased in the IR-shHMGB1-Exos group after IR (Fig. 4E). These results suggest that IR-shHMGB1-Exos increases ESCC apoptosis by affecting the expression levels of Bcl-2 and Bax after IR.

### IR-shHMGB1Exos induce G2/M arrest in ESCC cells after IR compared with IR-NC-Exos

Cell cycle detection is essential for understanding the mechanism by which exosomes affect the radiosensitivity of ESCC cells. Flow cytometry showed that KYSE150 and ECA109 cell cycle were arrested at the G2/M stage in both IR-NC-Exos group (P = 0.048, P = 0.012) and IR-shHMGB1-Exos group (P = 0.0001, P < 0.0001) after IR. The G2/M arrest of KYSE150 (P = 0.0202) and ECA109 (P = 0.0127) cells co-cultured with IR-shHMGB1-Exos was more obvious than that of IR-NC-Exos (Fig. 4C, D). The expression levels of cyclin-related proteins were examined by western blotting, and the results showed that Cyclin B1 and CDK1 expression levels were downregulated in the IR-shHMGB1-Exos group after IR compared with those in the IR-NC-Exos group (Fig. 4F). These results indicated that IR-NC-Exos could reverse irradiation-induced degradation of Cyclin B1 and CDK1, help to eliminate G2/M arrest and promote cell cycle progression, while IR-shHMGB1-Exos could arrest ESCC cells in G2/M phase by reducing the expression of Cyclin B1 and CDK1, which reversed radioresistance.

### Exosomal HMGB1 enhances DNA damage repair after IR by PI3K/AKT/FOXOA3 pathway activation

**Fig. 5:**
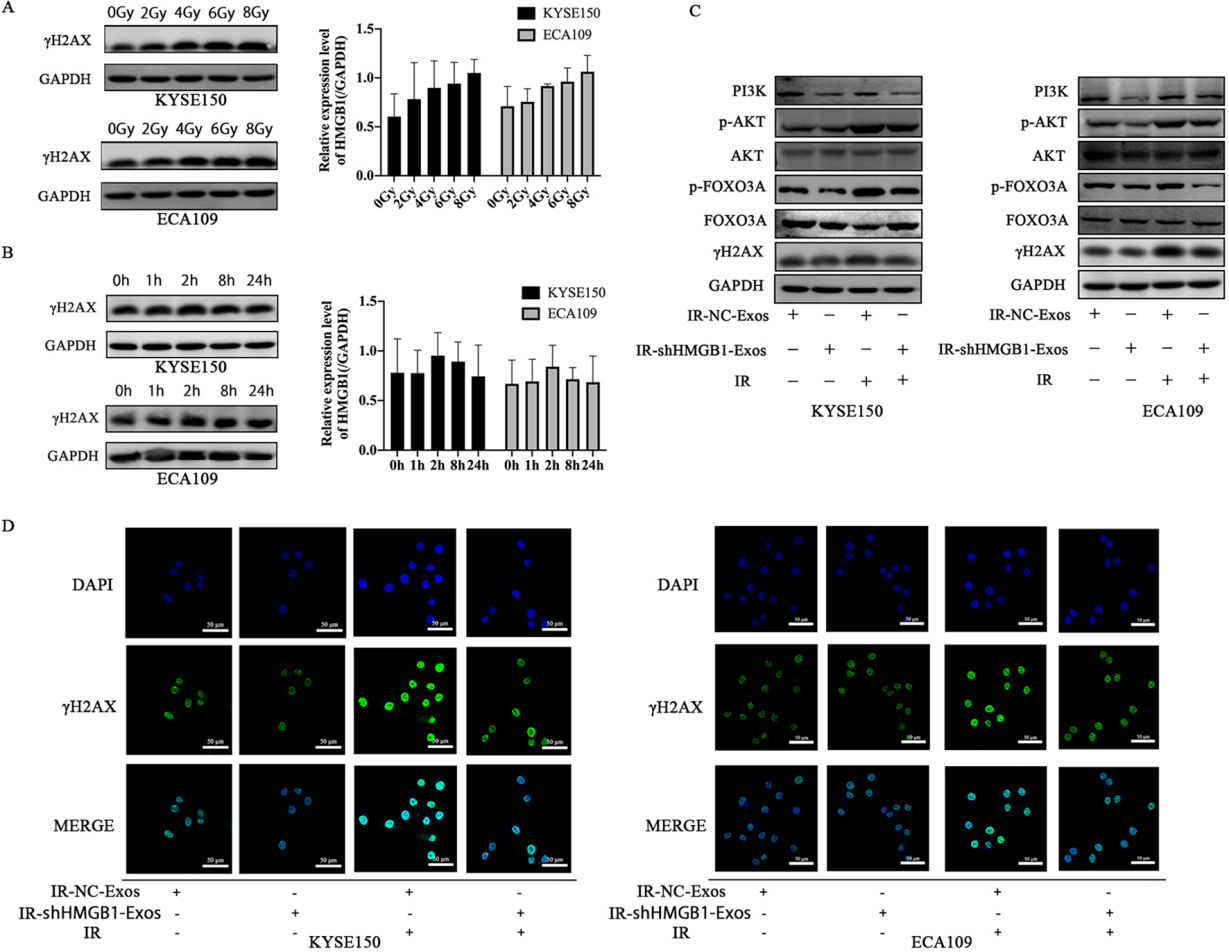
Exosomal HMGB1 played a carcinogenic role by activating PI3K/AKT/FOXO3A signaling pathway. **A** The protein expression of γH2AX varied with the radiation dose in cells. **B** The changes in γh2ax protein expression after 8 Gy IR were related to time. **C** Western blot was used to detect the protein expression of PI3K/pAKT/AKT/pFOXO3A/FOXO3A/γH2AX after exosomes treatment. **D** After treatment with different exosomes, the number of γH2AX focus formations was observed by immunofluorescence assay (magnification, ×600). HMGB1: high mobility group box 1; IR: irradiation

The γH2AX protein expression was positively correlated with the IR dose (Fig. 5A), and the expression level at 2 h after IR was greater than that at the other time points (Fig. 5B). Therefore, the subsequent experiments of rh2ax were carried out at 2 h after 8 Gy irradiation. We examined the changes in γH2AX after IR, the expression level of γH2AX in KYSE150 and ECA09 cells that were treated with different exosomes and the expression and localization of γH2AX in KYSE150 and ECA109 cells that were treated with the two kinds of exosomes by immunofluorescence analysis. The expression level of γH2AX in the IR-shHMGB1-Exos group was not different from that in the IR-NC-Exos group before IR (green fluorescence represents γH2AX). However, our results showed that the expression level of γH2AX in the two groups was significantly increased after IR (Fig. 5D). Compared with that in the IR-NC-Exos group, the ability of IR-shHMGB1-Exos to repair KYSE150 and ECA109 cells were reduced. γH2AX focal formation was significantly increased, especially in the IR-NC-Exos group (Fig. 5C). These findings suggest that IR-induced exosomal HMGB1 can regulate γH2AX protein expression and enhance DNA damage repair, which is involved in the radioresistance of ESCC cells.

**Fig. 6:**
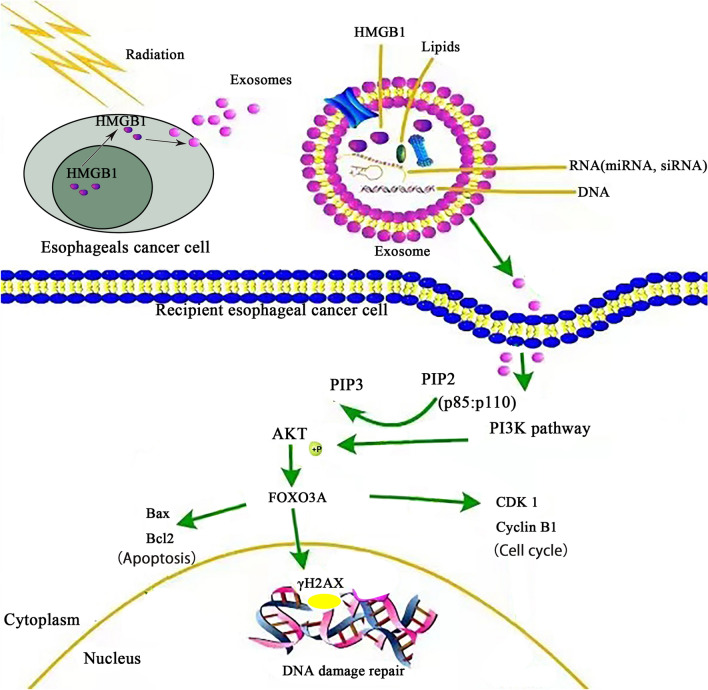
Irradiation-induced release of exosomal HMGB1, which is received by recipient cells and activates the PI3K-AKT-FOXO3A pathway mechanism diagram

Some research has highlighted the PI3K/AKT/FOXO3A signaling pathway as a target for tumor radioresistance. Therefore, we investigated whether this signaling pathway was affected by IR-induced exosomes processing. We examine the interaction between IR-induced exosomes and γH2AX and found that exosomal HMGB1 may activate the PI3K/AKT/FOXO3A signaling pathway. The results showed that the protein expression of PI3K/pAKT/pFOXO3A increased after IR-NC-Exos treatment with or without IR (Fig. 5C). The mechanism of Irradiation-induced release of exosomal HMGB1 activates the PI3K-AKT-FOXO3A pathway in receptor cells is shown in Fig. 6. These findings suggest that IR-induced exosomal HMGB1 confers radioresistance by activating the PI3K/AKT/FOXO3A signaling pathway.

## Discussion

Radiotherapy is a well-established therapeutic method to shrink tumor mass and eliminate tumor cells by damaging tumor cells through DNA disruption, ROS production and the destruction of cell structural integrity [28]. The radiosensitivity of different tumors is significantly different. However, resistance to IR is still a major obstacle in ESCC treatment [29]. Thus, extensive efforts should be made to investigate the potential targets and eliminate radioresistance.

Emerging evidence has indicated that serum-based exosomes can serve as important biomarkers of lung cancer [30], colorectal cancer [31], glioma [32], endometrial cancer [33], liver cancer [34], and prostate cancer [35]. In this study, the expression level of HMGB1 in the plasma exosomes of ESCC patients was higher than that of healthy individuals, and the radioresistance of ESCC patients with high HMGB1 expression (n = 11) was significantly higher than that of ESCC patients with low HMGB1 expression (n = 10). Therefore, the expression level of HMGB1 in plasma exosomes can be used as a minimally invasive diagnostic marker to predict the radiosensitivity of ESCC. In addition, recent studies have shown that exposing various cells to environmental stress can cause tumor cells to increase the release of exosomes and change their molecular composition [36]. IR can damage DNA and other structures in target cells, which is a stress condition that affects the biological behavior of tumor cells [37]. IR-induced exosomes are released to participate in transcription, translocation and cell division [38–40].

IR-induced the bystander effect (RIBE) in which exosomes can influence the function of recipient cells that have not been exposed to IR [41, 42]. Currently, there is no consensus on the role of exosomes in cancer cell proliferation and radiosensitivity [43, 44]. Recently, Yamana et al. found no change in the proliferation of OSCC cells treated with exosomes induced by radiation, but increased the radiation resistance of radiation sensitive cells [43]. Payton et al. reported that exosomes derived from radiation resistant cells in breast cancer can increase cell viability and colony formation in recipient cells and increase resistance to chemotherapy and radiotherapy [45]. In this study, we found that exosomes from irradiated donor cells could promote the proliferation and radioresistance of ESCC cells. In addition, ESCC cells treated with irradiated exosomes potentially have a higher ability for radiosensitivity regulation than that of ESCC cells treated with non-irradiated exosomes. These results suggest that IR-Exos may act specifically at sites where cells are irradiated and some damage occurs.

Our previous study reported that HMGB1 expression was frequently upregulated in ESCC tissue and negatively related to improved clinical long-term outcomes; HMGB1 expression promotes DNA damage repair and confers radioresistance [26]. In this study, we confirmed that HMGB1 was released by exosomes into the extracellular space after IR and was absorbed by other cells to promote DNA damage repair, leading to radioresistance. Tang et al. revealed that HMGB1 could be released from stressed cells in the form of exosomes and was an important component of the tumor microenvironment after chemotherapy or radiotherapy [46]. Moreover, exosome-derived HMGB1 can significantly promote proliferation [47, 48], metastasis [49], immune escape [46], and radioresistance. Our results were consistent with previous studies. The results indicated that exosomes from irradiated donor cells could enhance the vitality of ESCC cells and significantly increase their radioresistance. In addition, HMGB1 knockdown could reduce the level of HMGB1 released by ESCC cells through exosomes after IR and reverse radioresistance.

The PI3K/AKT pathway is a commonly mutated oncogenic pathway in ESCC that acts as a key regulator of radioresistance [50]. Exosomes released from gastric cancer cells can activate the PI3K/AKT and MAPK/ERK signaling pathways, which are closely related to radiological resistance and tumor progression and ultimately promote the proliferation of recipient gastric cancer cells [51]. Furthermore, the PI3K/AKT/FOXO3A signaling pathway plays an important regulatory role in a variety of tumorigenic processes [52]. FOXO3A is a transcription factor that is downstream of AKT and can be inhibited by AKT-mediated phosphorylation. Activation of this pathway reduces the transcription of key genes that mediate a variety of cellular processes, including proliferation, cell cycle arrest, apoptosis and DNA damage [53, 54]. Interestingly, our study showed that IR-shHMGB1-Exos inhibited AKT phosphorylation in vitro, which indicated that IR-shHMGB1-Exos could reverse radioresistance in ESCC cells. These findings raised the possibility that targeting these signaling pathways may improve radioresistance.

Cell cycle dysregulation has been implicated in the proliferation of cancer cells, and irreversible cell cycle arrest may result in cellular senescence [55, 56]. Our results indicated that the IR-shHMGB1-Exos group had significantly increased cell cycle arrest in the G2/M phase after IR. Moreover, IR-shHMGB1-Exos treatment increased the number of cells that were arrested in G2/M phase, which was associated with the downregulation of cell cycle-related proteins, such as cyclin B1 and CDK1. This cell cycle arrest may trigger radioresistance by IR-shHMGB1-Exos. In addition, we found that the level of apoptosis in the IR-shHMGB1-Exos group was significantly higher than that in the negative control group. We hypothesize that the loss of HMGB1 in IR-Exos can reverse the apoptosis inhibition mediated by IR-induced exosomes. Bax and Bcl2 are apoptosis-related proteins that are implicated in radiosensitivity. Bax increases the formation of oligomers that participate in apoptogenic molecule release and initiate intrinsic apoptosis [57]; Bcl-2 proteins are a family of structurally related proteins that act as intrinsic pathway regulators of apoptosis [58]. The western blotting results revealed that Bax expression was upregulated with the downregulation of Bcl2 expression in the IR-shHMGB1-Exos group. Therefore, IR-induced exosomal HMGB1 inhibits apoptosis in irradiated ESCC cells by regulating members of the proapoptotic Bcl-2 family. However, the exact mechanism of the pathways in the IR-shHMGB1-Exos group that lead to apoptosis remains unclear and requires further investigation.

DNA damage repair (DDR) is one of the major modulators that confers radiosensitivity to tumor cells [59]. The γH2AX protein was shown to be an indicator of DNA double-strand breaks (DSBs) [60]. Exosomes derived from tumor cells increased the radioresistance of adjacent tumor cells by inducing the repair of DSBs [61]. HMGB1 binds to RAGE and TLR4 to promote the phosphorylation of PI3K/AKT and the expression of γH2AX [24]. Our study identified that ESCC cells release exosomal HMGB1 after IR, which regulates the phosphorylation and translocation of ATM by activating the PI3K/AKT/FOXO3A signaling pathway in recipient ESCC cells. Exosomal HMGB1 enhanced DDR in recipient cells and made tumor cells more resistant to radiotherapy. In addition, γH2AX expression in the IR-shHMGB1-Exos group was lower than that in the IR-NC-Exos Group 2 h after 8 Gy IR. These findings suggest that IR-shHMGB1-Exos resulted in a defective DDR, which improved radiosensitivity.

## Conclusion

In conclusion, our study demonstrates that exosomes derived from irradiated donor cells trigger the PI3K-AKT-FOXO3A signaling pathway in recipient cancer cells and induce a radioresistant phenotype. IR-induced exosomal HMGB1 is a promising clinical target for overcoming radioresistance in ESCC.

## Data Availability

The datasets used and/or analyzed during the current study are available from the corresponding author on reasonable request.

## Abbreviations

EC: Esophageal cancer
ESCC: Esophageal squamous cell carcinoma
HP: Healthy people
HMGB1: High mobility group box 1
Exos: Exosomes
IR: Irradiation
γ-H2AX: Phosphorylated histone H2AX
DDR: DNA damage repair
DSBs: Double-strand breaks
FBS: Fetal bovine serum
NC: Negative control
RT-q PCR: Real-time quantitative polymerase chain reaction
SER: Sensitizer enhancement ratios
TBST: TBS containing 0.1% Tween 20.
